# A comparative study of traditional high-fidelity (manikin-based) simulation and virtual high-fidelity simulations concerning their effectiveness and perception

**DOI:** 10.3389/fmed.2025.1523768

**Published:** 2025-02-10

**Authors:** Izabela Sałacińska, Patrycja Trojnar, Krisztina Éles Gebriné, Viktória Törő, Attila Sárváry, Paweł Więch

**Affiliations:** ^1^Faculty of Health Sciences and Psychology, Collegium Medicum, University of Rzeszów, Rzeszów, Poland; ^2^Institute of Health Care, Academy of Applied Sciences, Przemyśl, Poland; ^3^Department of Nursing and Midwifery, Faculty of Health Sciences, University of Debrecen, Nyíregyháza, Hungary; ^4^Doctoral School of Health Sciences, University of Debrecen, Nyíregyháza, Hungary; ^5^Department of Integrative Health Sciences, Faculty of Health Sciences, University of Debrecen, Nyíregyháza, Hungary

**Keywords:** simulation, educational method, effectiveness, medical students, soft skills, high fidelity simulation

## Abstract

**Introduction:**

Medical simulation has become an integral part of medical student education. There is a limited body of literature comparing virtual and high-fidelity simulation in terms of effectiveness and student perception.

**Methods:**

A total of 130 medical students at the University of Rzeszów participated in this cross-sectional study. The respondents were divided into two groups: students who completed a selected scenario using a virtual patient (Body Interact) and students who completed a scenario using traditional high-fidelity (manikin-based) simulation (HFS). After completing the scenario, students filled in the following questionnaires: the Simulation Design Scale (SDS), the Educational Practices Questionnaire (EPQ), the Student Satisfaction and Self-Confidence in Learning Scale (SSCL) and a customized survey questionnaire.

**Results:**

The study found no significant difference in the effectiveness of HFS between students exposed to either type of simulation. Detailed analysis within specific categories – problem-solving, teamwork, and active learning – also showed no significant differences between virtual and traditional HFS. Furthermore, there were no notable differences between virtual and traditional simulations regarding specific aspects such as satisfaction with learning, self-confidence in learning, and expectations. However, within the virtual simulation group, females rated active learning significantly higher. Students aged 24–33 rated satisfaction with learning, self-confidence, overall effectiveness and perception of HFS, problem-solving, and active learning more favorably. Additionally, the levels of perceived effectiveness and satisfaction of higher years students with HFS increased.

**Conclusion:**

Virtual patient simulation and traditional HFS foster the development of practical skills, as well as soft skills of medical students in challenging situations.

## Introduction

Significant technological advancement led the development and widespread use of simulation-based training in the education (1–4). Simulation-based training (SBT), such as high-fidelity simulation (HFS), has become an integral part of undergraduate healthcare education. It has been incorporated into medical curricula (1–4). High-fidelity simulation provides a safe learning and immersive clinical environment for students to integrate and apply their theoretical and practical knowledge (2). This type of simulation allows students to practice both technical and non-technical skills (communication, teamwork, decision making) (5). One of the main aims of high-fidelity simulation is to prepare students for the clinical practice (2, 3). In particular, SBT has been integrated into schools of nursing and medicine and has been associated with higher satisfaction (6).

The effectiveness of the SBT and HFS has been investigated in terms of knowledge acquisition, self-efficacy, satisfaction, confidence and various non-technical skills (1–8). A meta-analysis investigated the effect of SBT on airway management training. It was found that SBT improved behavioral performance, but there was no significant change in time skill, written examination score and success rate of completing procedures on patients (7). The most recent meta-analysis showed that SBT significantly improved the theoretical scores, skill scores and non-technical skills of medical students, interns and residents in anesthesia (8). However, it should be noted that there was heterogeneity in the results of the studies.

The online form of training used during COVID-19 showed how important the methods of transferring knowledge are and how this influences the perception of students (9, 10). Perception, involving the active reception, analysis and interpretation of sensory phenomena, is a process whereby current information is processed based on registered information (9, 10). Students’ perceptions of the medical education environment can have a direct impact on their level of engagement in learning (9, 10). Self-regulatory learning skills are associated with an indirect effect of perception of the medical education environment on academic engagement (9). It is worth noting that both efficiency and perception are processes that are susceptible to individual differences (9). Learning effectiveness and efficiency also depend on the learner’s experience, knowledge and personal dispositions (9, 11). These are important areas of research related to human thinking and cognitive processes (9, 11). SBT and high-fidelity simulation can improve the knowledge, inter-professional collaboration, confidence, critical thinking and clinical skills of both medical and nursing students (12–18). A meta-analysis found that HFS was more effective than other teaching methods in increasing knowledge, skills, cooperation, caring and interest in learning among undergraduate nursing students (18). The findings of this study lend further credence to the notion that high-fidelity simulation is an effective teaching method in comparison with other traditional or novel techniques. Simulation-based training (SBT) is thus considered a valuable learning method for healthcare students and qualified healthcare professionals alike. A further systematic review has indicated that SBT is a useful tool for the improvement of human factor skills in qualified healthcare teams, with the potential to contribute to an enhancement of patient safety (19).

In the context of the educational process, it is imperative to recognize the importance of students deriving satisfaction from the learning experience (20). It improves student engagement and commitment and can contribute to more effective acquisition of knowledge and skills (20). Therefore, it is important to measure satisfaction and confidence in learning as an outcome of learning (21). Confidence is influenced by many factors including personality, experience, expectations, social and cultural conditioning (22). Several studies investigated how HFS influences the self-confidence and satisfaction of the health care students (12–18). A previous study measured the confidence and satisfaction of medical students and reported an improvement in both areas. There was also a strong correlation between ratings of the learning experience and both satisfaction and confidence. The article highlights the importance of well-designed teaching methods and skilled teachers (17).

The effectiveness of HFS was investigated in many studies from different aspects (17–19). In the available medical database, limited number of articles can be found regarding the comparison of effectiveness and perception between mannequin high-fidelity simulation and simulation using a virtual patient. The aim of the present study was twofold: firstly, to analyze and assess the effectiveness of the traditional high-fidelity (manikin-based) simulation in comparison with the virtual patient simulation; and secondly, to consider the perception of these simulations in the educational process for medical students.

## Methods

### Ethical statements

The study was conducted in accordance with ethical standards laid down in an appropriate version of the Declaration of Helsinki and Polish national regulations. The study was approved by the institutional Bioethics Committee at the University of Rzeszów (Resolution No. 2023/04/0022 on 5 April 2023). The participants, who consented to participate in the study, were informed of the purpose of the study, and were able to withdraw from the study at any stage without giving reasons.

### Study design

The study was cross-sectional, observational conducted under simulated conditions. The study was conducted at the Medical Simulation Centre of the University of Rzeszow (UR). A Susie Gaumard high-fidelity simulator and Body Interact (BI) virtual patient software were used to achieve the study objective.

### Research questions and hypothesis

The following research questions were identified to address the aim of the study

1. What is the difference in the effectiveness between high-fidelity (manikin-based) and virtual patient simulation in the study group of medical students?
2. What is the difference in the perception between high-fidelity (manikin-based) and virtual patient simulation in the study group of medical students?
3. Do variables such as gender, age and year of study significantly differentiate the effectiveness and perception of medical students in the training process?

Based on the above research problems, the following research hypotheses were identified:

1. It is hypothesized that the use of traditional high fidelity (manikin-based) simulation will increase its effectiveness, as represented by teamwork, active learning and problem solving, to a greater extent than virtual simulation.
2. It is hypothesized that the use of traditional high fidelity (manikin-based) simulation raises the satisfaction, confidence and expectations of the medical students surveyed to a greater extent, in relation to virtual simulation.
3. Selected variables significantly influence differences in the perception and effectiveness of high-fidelity simulation in a group of medical students.

### Subjects

**Figure 1:**
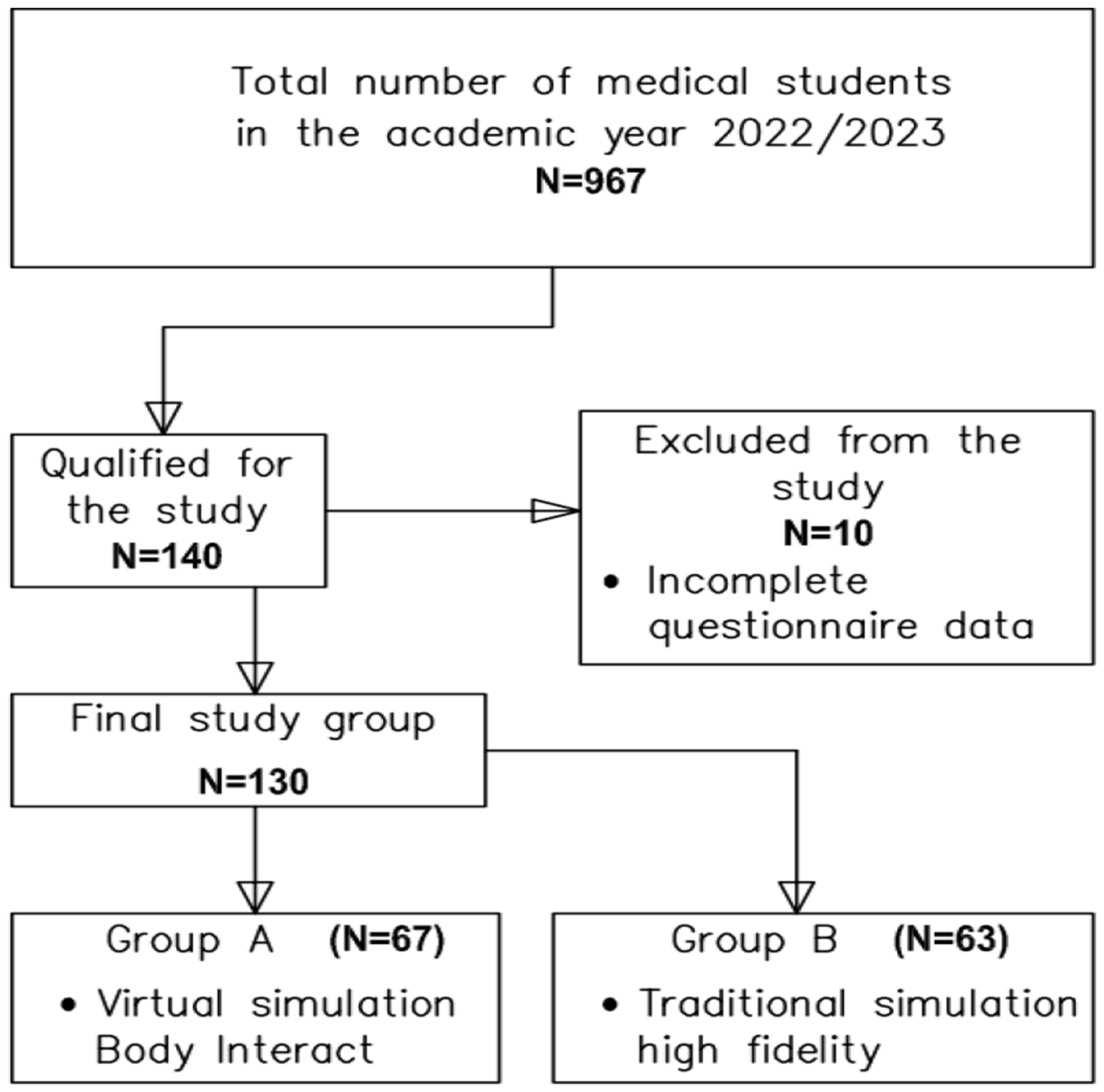
Flow chart demonstrating study participants selection.

A total of 130 medical students from the University of Rzeszów (UR), all at least in their second year of study, participated in the research. During the study, respondents were assigned to two groups. Group A consisted of students who completed a specific scenario using the virtual patient BI. Group B included students who engaged in a scenario within a traditional high fidelity (manikin-based) simulation. A flow chart demonstrating selection of the study group is presented in Figure 1.

In order to ensure the reliability of the statistical analyses, 130 subjects out of a total of 967 medical students from the University of Rzeszów were included in the study.

This institution is the only one in the Podkarpackie Voivodeship training future physicians. It also participated in the International Body Interact Project, which facilitated remote learning during the SARS-CoV-2 pandemic, as the sole university in Poland (23). In addition, the University of Rzeszów operates a Medical Simulation Centre (MSC), which has high-fidelity simulators with features that enable a wide range of research to be carried out.

The selection of the number of students for the study, based on the total number of students in the program, is shown in Table 1. Before starting the main study, a pilot test was conducted with a sample of 50 medical students from the University of Rzeszów. Due to the use of two types of simulation, 24 students were tested with virtual simulation using Body Interact, while 26 students participated in traditional high fidelity (manikin-based) simulation. At this stage, no concerns were identified regarding the understanding of the questions contained in the survey questionnaire or the overall design of the questionnaire.

**Table 1:** Selection of the number of students for the study.

Number of students for the academic year 2022/2023	
Year of Study	Medical students
2	212
3	201
4	191
5	183
6	180
TOTAL (POPULATION)	967
Maximum allowable error	(+/−5%) for the confidence level
Total	121

According to the established guidelines, the minimum sample size was calculated to be 121 participants. The following general formula for minimum sample size was applied:


$$\mathrm{Nmin}\;\cdot \;\left(E2\;\cdot \;N+\alpha 2\;\cdot \;p\;\cdot \;\left(1-p\right)\right)=N\;\cdot \;\alpha 2\;\cdot \;p\;\cdot \;\left(1-p\right)$$

where: *N* stands for the population size, α is the significance level, *p* is the proportion of the population and E is the maximum allowable error.

The minimum sample size was calculated with a confidence level of 95%, a maximum error of 5%, and a fraction size of 90%. The total population of medical students was 967, and the calculated minimum sample size for the study was 121 participants.

To account for potential data loss of up to 4% due to incomplete or incorrectly completed surveys, the minimum sample size was increased from 121 to 140 participants. Participants were selected by the researcher (purposive sampling). After careful review, 10 of the 140 completed questionnaires were excluded because they did not meet the inclusion criteria for the study. This resulted in 130 fully completed questionnaires that met the requirements of the study.

Inclusion criteria for the study included students who were at least in their second year of medical school, had successfully completed coursework in physical examination, had experience with medical simulation activities, and had consented to participate in the study. Participation was voluntary and anonymous. Each respondent received a questionnaire along with instructions and an explanation of the purpose of the study.

Among the 130 participants, 67 students (51.5%) underwent virtual simulation using Body Interact, while 63 students (48.5%) participated in traditional high fidelity (manikin-based) simulation. The characteristics of the study group are shown in Table 2. Among the participants, there were 83 women (63.9%) and 47 men (36.2%). Seventy-seven individuals (59.2%) were aged 19–23 years, while 53 individuals (40.8%) were aged 24–33 years. Significant differences were observed in the educational levels of students between the two groups (*p* < 0.001). In the traditional high fidelity (manikin-based) simulation group, the participants were predominantly fourth-year students and above.

**Table 2:** Characteristics of the studied group.

		Virtual simulation body interact		Traditional HF simulation		Total	
		*N*	%	*N*	%	*N*	%
Gender	Women	38	56.7%	45	71.4%	83	63.9%
	Men	29	43.3%	18	28.6%	47	36.2%
Age	19–23 y.	36	53.7%	41	65.1%	77	59.2%
	24–33 y.	31	46.3%	22	34.9%	53	40.8%
Year of study	Second	24	35.8%	0	0.0%	24	18.5%
	Third	23	34.3%	0	0.0%	23	17.7%
	Fourth	6	9.0%	43	68.3%	49	37.7%
	Fifth	12	17.9%	9	14.3%	21	16.2%
	Sixth	2	3.0%	11	17.5%	13	10.0%

HF, high-fidelity.

The study used Gaumard’s Susie S2000 Advanced Nursing and Emergency High-Fidelity Simulator. The Susie simulator is wireless and is designed to provide effective and realistic simulation learning experiences for students at all levels (24). Conversely, the Body Interact (BI) virtual patient simulator from Take the Wind Ltd. was available to the researcher in a portable format. The BI software provides access to a variety of scenarios covering medical cases related to pre-hospital care, emergency department situations and inpatient care. Training includes learning to make accurate diagnoses and initiate therapy, analyzing and interpreting data from patient monitoring devices, performing virtual physical examinations, and many other functions specific to the selected scenario (25).

### Assessments and research tools

Group A students were introduced to a similar sample scenario in the Body Interact virtual reality tool, where they familiarized themselves with the technical aspects of the simulation tool, before participating in the actual research scenario. Students assigned to Group B were familiarized with the simulation environment (room, equipment) and the Susie S2000 simulator. Selection into Group A or B was determined by the researcher. The Body Interact software was available to the researcher on Wednesdays, Thursdays and Fridays; while the medical students (all year groups) had classes at MCS every day from Monday to Friday. Each student who consented to participate in the study was only allowed to take part once and only in one type of simulation depending on the availability of equipment on a given day.

Students in both Group A and B engaged in the same scenario addressing life-threatening conditions in adults across both types of simulations. The study was conducted as a part of supplementary classes on medical simulation, in which the researcher implemented the same simulation scenario with each group of students using both traditional high fidelity (manikin-based) simulation and virtual reality. Students in Group A performed a scenario with software using a virtual patient in a specific BI scenario. The students had the opportunity to take a history, perform a physical examination, administer medication or perform medical interventions. Group B students, on the other hand, carried out the same scenario as group A students, working on a Susie 2000 high-fidelity simulator. They also had the opportunity to conduct a history and physical examination, administer medication or perform specialized procedures.

The study was conducted as part of a supplementary medical simulation course, where the researcher ran the same simulation scenario with each group of students, using both traditional high-fidelity (manikin-based) simulation and virtual reality. The first part of the simulation for each group consisted of a 10-min pre-briefing during which the students were introduced to the simulation environment, including the equipment present in the room and the simulator and its functions. This part covered the objectives of the simulation, the rules and assumptions, and the distribution of roles among the group members. The students then performed the simulation scenario for 10 min. After completion of the scenario, a debriefing session was held to discuss the content of the scenario. This debriefing consisted of four phases: the emotional phase, the descriptive phase, the analytical phase and the application phase, and lasted 25 min in total. The intention of the debriefing was to help participants process any distressing experiences they may have had, to provide an account of what happened during the scenario, and to encourage a discussion of the positive and negative actions that occurred. The aim was to identify solutions that would be constructive in addressing the challenges faced during the exercise. Following the debrief, students were asked to complete an electronic questionnaire, with assurances of anonymity and that the results would only be used for the stated research purpose.

Three standardized instruments and an author-developed questionnaire were used in the study. The SSCL instrument was used to assess the level of student satisfaction and confidence in the medical simulation-based learning process. It also allows the assessment of the level of efficiency. The first section of the SSCL contains five questions about student satisfaction with the educational process, while the second section contains eight questions about confidence in the learning process (26). The Simulation Design Scale – Student Version (SDS) was used to assess specific features of the simulation and their importance to students. The first section of the SDS contains five statements related to objectives and information, the second section contains questions to assess support, the third section is about problem solving, the fourth section is about reflection with feedback and the fifth section contains questions related to fidelity (27). The Educational Practices Questionnaire (EPQ) was used to assess the validity of the educational techniques used. This tool consists of 16 statements divided into four sections (subscales): active student learning during the simulation process, teamwork collaboration during the simulation, different learning methods and student expectations during the scenario execution (28).

### Statistical analysis

Statistical analysis of quantitative variables was performed using the Mann–Whitney *U* test. The normality of the distributions of the examined variables was assessed using the Shapiro–Wilk test, while the homogeneity of variance was evaluated using Levene’s test. A significance level of *p* < 0.05 was accepted. Quantitative variables included all variables except sociodemographic data. The assumptions of the survey were not compromised. The statistical analysis was conducted using Statistica 13.3 TIBCO software.

## Results

### Effectiveness of high-fidelity simulation in the study group of medical students depending on the method used

There was no difference in the effectiveness of high-fidelity simulation between students in both types of simulation (*p* = 0.355). No significant differences were found between virtual and traditional high fidelity (manikin-based) simulation when considering detailed results in the specified categories, namely problem-solving, teamwork, and active learning (Table 3). The subscales used for the calculations were: Satisfaction with learning, Self-Confidence in learning, Expectations, Perception of high-fidelity simulation.

**Table 3:** Effectiveness of high-fidelity (HF) simulation.

Effectiveness of high-fidelity simulation (1–5 points)	Virtual simulation body interact					Traditional HF simulation					Z	*p*-value
	*M*	Me	Min	Max	SD	M	Me	Min	Max	SD		
Problem-solving	4.21	4.40	1.00	5.00	0.81	4.44	4.60	1.00	5.00	0.66	−1.55	0.120
Team collaboration	4.72	5.00	3.00	5.00	0.60	4.73	5.00	1.00	5.00	0.70	0.02	0.987
Active learning	4.46	4.80	2.00	5.00	0.69	4.57	4.70	1.00	5.00	0.64	−0.60	0.548
Effectiveness of high-fidelity simulation	4.47	4.63	2.73	5.00	0.58	4.58	4.70	1.00	5.00	0.59	−0.92	0.355

*Z – Mann–Whitney U test value; p – test probability indicator; M, mean; Me, median; SD, standard deviation.*

### Perception of high-fidelity simulation in the study group of medical students depending on the method used

The perception of high-fidelity simulation did not differ among students subjected to different types of simulation (*p* = 0.289). No significant differences were observed between virtual and traditional high fidelity (manikin-based) simulation when considering detailed results in the specified categories, namely satisfaction with learning, self-confidence in the learning process, and expectations (Table 4). The subscales used for the calculations were: Problem solving, teamwork collaboration during simulation, active student learning during the simulation process, Effectiveness of high-fidelity simulation.

**Table 4:** Perception of high-fidelity (HF) simulation.

Perception of high-fidelity simulation (1–5 points)	Virtual simulation body interact					Traditional HF simulation					*Z*	*p*-value
	M	Me	Min	Max	SD	M	Me	Min	Max	SD		
Satisfaction with learning	4.35	4.60	2.60	5.00	0.64	4.45	4.60	1.00	5.00	0.70	−1.18	0.239
Self-confidence in the learning process	4.21	4.13	2.63	5.00	0.64	4.35	4.50	1.00	5.00	0.63	−1.40	0.161
Expectations	4.54	5.00	2.50	5.00	0.68	4.57	5.00	1.00	5.00	0.74	−0.29	0.771
Perception of High-Fidelity Simulation	4.37	4.51	2.78	5.00	0.59	4.46	4.65	1.00	5.00	0.64	−1.06	0.289

*Z – Mann–Whitney U test value; p – test probability indicator; M, mean; Me, median; SD, standard deviation.*

### Perception and effectiveness of high-fidelity simulation with respect to gender, age and year of study of medical students

They were compared between the two groups, taking into account factors such as gender, age of the respondents, and year of study. The results of the analysis of differences between the groups are presented in Table 5, with detailed breakdowns provided in Tables 6–9.

**Table 5:** Perception and effectiveness of high-fidelity (HF) simulation.

	Virtual simulation body interact						Traditional HF simulation					
	Gender		Age		Year of study		Gender		Age		Year of study	
	*Z*	*p*	*Z*	*p*	*Z*	*p*	*Z*	*p*	*Z*	*p*	*Z*	*p*
Satisfaction with learning	1.34	0.180	**−2.17**	**0.030**	**−3.63**	**<0.001**	**2.40**	**0.017**	0.18	0.859	–	–
Self-confidence in the learning process	1.01	0.312	**−2.68**	**0.007**	**−3.15**	**0.002**	1.13	0.257	−1.12	0.264	–	–
Expectations	0.22	0.824	−1.50	0.134	**−2.10**	**0.036**	0.81	0.416	−0.85	0.394	–	–
Perception of high-fidelity simulation	0.93	0.352	**−2.61**	**0.009**	**−3.50**	**<0.001**	1.59	0.111	−0.61	0.539	–	–
Problem-solving	1.56	0.118	**−2.85**	**0.004**	**−3.58**	**<0.001**	**2.14**	**0.033**	−0.14	0.888	–	–
Team collaboration	0.76	0.449	−1.55	0.121	−1.54	0.123	−0.58	0.565	−0.56	0.579	–	–
Active learning	**1.97**	**0.049**	**−2.15**	**0.032**	**−2.23**	**0.025**	1.48	0.139	−0.99	0.323	–	–
Effectiveness of high-fidelity simulation	1.54	0.125	**−2.98**	**0.003**	**−3.17**	**0.002**	1.49	0.137	−0.54	0.586	–	–

*Z* – Mann–Whitney *U* test value; *p* – test probability indicator. Significant findings (*p* < 0.05) are bolded.

**Table 6:** Perception and effectiveness of high-fidelity simulation by gender in the group subjected to virtual simulation body interact.

	Women					Men					*Z*	*p*-value
	M	Me	Min	Max	SD	M	Me	Min	Max	SD		
Satisfaction with learning	4.44	4.60	2.80	5.00	0.60	4.23	4.40	2.60	5.00	0.68	1.34	0.180
Self-confidence in the learning process	4.28	4.38	2.63	5.00	0.63	4.12	4.13	2.75	5.00	0.66	1.01	0.312
Expectations	4.59	5.00	2.50	5.00	0.64	4.48	5.00	3.00	5.00	0.74	0.22	0.824
Perception of high-fidelity simulation	4.44	4.58	2.84	5.00	0.56	4.28	4.42	2.78	5.00	0.64	0.93	0.352
Problem-solving	4.32	4.80	1.00	5.00	0.86	4.08	4.20	2.60	5.00	0.74	1.56	0.118
Team collaboration	4.80	5.00	3.00	5.00	0.47	4.62	5.00	3.00	5.00	0.74	0.76	0.449
Active learning	**4.55**	5.00	2.00	5.00	0.71	4.35	4.60	3.00	5.00	0.65	**1.97**	**0.049**
Effectiveness of high-fidelity simulation	4.56	4.85	2.73	5.00	0.54	4.35	4.53	3.07	5.00	0.62	1.54	0.125

*Z* – Mann–Whitney *U* test value; *p* – test probability indicator; M, mean; Me, median; SD, standard deviation. Significant findings (*p* < 0.05) are bolded.

**Table 7:** Perception and effectiveness of high-fidelity simulation by age in the group subjected to virtual simulation body interact.

	Upto 23 years old					Upto 33 years old					*Z*	*p*-value
	M	Me	Min	Max	SD	M	Me	Min	Max	SD		
Satisfaction with learning	4.21	4.20	2.80	5.00	0.64	**4.52**	4.60	2.60	5.00	0.60	**−2.17**	**0.030**
Self-confidence in the learning process	4.03	4.13	2.63	5.00	0.62	**4.42**	4.63	2.75	5.00	0.62	**−2.68**	**0.007**
Expectations	4.44	4.75	2.50	5.00	0.73	4.66	5.00	3.00	5.00	0.60	−1.50	0.134
Perception of high-fidelity simulation	4.23	4.31	2.84	5.00	0.59	**4.53**	4.77	2.78	5.00	0.57	**−2.61**	**0.009**
Problem-solving	3.97	3.90	1.00	5.00	0.83	**4.50**	4.80	2.60	5.00	0.71	**−2.85**	**0.004**
Team collaboration	4.61	5.00	3.00	5.00	0.71	4.85	5.00	3.00	5.00	0.43	−1.55	0.121
Active learning	4.30	4.50	2.00	5.00	0.75	**4.66**	5.00	3.10	5.00	0.56	**−2.15**	**0.032**
Effectiveness of high-fidelity simulation	4.29	4.38	2.73	5.00	0.60	**4.67**	4.93	3.17	5.00	0.49	**−2.98**	**0.003**

*Z* – Mann–Whitney *U* test value; *p* – test probability indicator; M, mean; Me, median; SD, standard deviation. Significant findings (*p* < 0.05) are bolded.

**Table 8:** Perception and effectiveness of high-fidelity simulation by year of study in the group subjected to virtual simulation body interact.

	2nd–3rd year of studies					4th–6th year of studies					*Z*	*p*-value
	M	Me	Min	Max	SD	M	Me	Min	Max	SD		
Satisfaction with learning	4.17	4.20	2.60	5.00	0.66	**4.76**	5.00	3.80	5.00	0.35	**−3.63**	**<0.001**
Self-confidence in the learning process	4.05	4.13	2.63	5.00	0.64	**4.59**	4.81	3.63	5.00	0.47	**−3.15**	**0.002**
Expectations	4.44	5.00	2.50	5.00	0.74	**4.80**	5.00	4.00	5.00	0.41	**−2.10**	**0.036**
Perception of high-fidelity simulation	4.22	4.33	2.78	5.00	0.62	**4.72**	4.87	3.81	5.00	0.36	**−3.50**	**<0.001**
Problem-solving	3.99	4.00	1.00	5.00	0.85	**4.74**	5.00	4.00	5.00	0.37	**−3.58**	**<0.001**
Team collaboration	4.64	5.00	3.00	5.00	0.69	4.93	5.00	4.00	5.00	0.24	−1.54	0.123
Active learning	4.34	4.60	2.00	5.00	0.74	**4.77**	5.00	3.50	5.00	0.44	**−2.23**	**0.025**
Effectiveness of high-fidelity simulation	4.32	4.40	2.73	5.00	0.61	**4.81**	4.93	3.93	5.00	0.29	**−3.17**	**0.002**

*Z* – Mann–Whitney *U* test value; *p* – test probability indicator; M, mean; Me, median; SD, standard deviation. Significant findings (*p* < 0.05) are bolded.

**Table 9:** Perception and effectiveness of high-fidelity simulation by gender in the group subjected to traditional simulation.

	Women					Men					*Z*	*p*-value
	M	Me	Min	Max	SD	M	Me	Min	Max	SD		
Satisfaction with learning	**4.57**	4.80	2.20	5.00	0.58	4.16	4.40	1.00	5.00	0.89	**2.40**	**0.017**
Self-confidence in the learning process	4.43	4.50	3.00	5.00	0.50	4.17	4.31	1.00	5.00	0.88	1.13	0.257
Expectations	4.63	5.00	3.00	5.00	0.60	4.42	4.75	1.00	5.00	1.02	0.81	0.416
Perception of high-fidelity simulation	4.54	4.68	3.32	5.00	0.48	4.25	4.41	1.00	5.00	0.91	1.59	0.111
Problem-solving	**4.56**	4.80	3.00	5.00	0.50	4.13	4.20	1.00	5.00	0.90	**2.14**	**0.033**
Team collaboration	4.74	5.00	2.00	5.00	0.58	4.69	5.00	1.00	5.00	0.96	−0.58	0.565
Active learning	4.67	4.90	3.50	5.00	0.42	4.31	4.60	1.00	5.00	0.97	1.48	0.139
Effectiveness of high-fidelity simulation	4.66	4.83	3.67	5.00	0.40	4.38	4.63	1.00	5.00	0.91	1.49	0.137

*Z* – Mann–Whitney *U* test value; *p* – test probability indicator; M, mean; Me, median; SD, standard deviation. Significant findings (*p* < 0.05) are bolded.

No differences were observed in the results obtained in the traditional high fidelity (manikin-based) simulation group with regard to the age of the students. Similarly, regarding the year of study, no differences were observed and no differences were calculated (taking into account the division between 2nd and 3rd year versus 4th to 6th year), as this group only included students in years 4–6 (Table 5). In the results obtained, it should be taken into account that mainly senior students (4th-6th year) participated in traditional high fidelity (manikin-based) simulation which may affect the results obtained and be a limitation of this study. The subscales used in the calculations were Satisfaction with learning, confidence in learning, expectations, perception of high-fidelity simulation, problem solving, teamwork, active learning, effectiveness of high-fidelity simulation.

Results revealed that women in the virtual simulation group rated their satisfaction with learning and problem – solving significantly higher than men (*p* = 0.049) (Table 6). The subscales used for the calculations were: Satisfaction with learning, Confidence in learning, Expectations, Perception of high-fidelity simulation, Problem-solving, Team collaboration, Active learning, Effectiveness of high-fidelity simulation.

Analyzing the age of students subjected to virtual simulation, it was found that students aged 24–33 rated their satisfaction with learning, self-confidence, overall perception of high-fidelity simulation, problem-solving, active learning, and the overall effectiveness of high-fidelity simulation more positively (Table 7). It should be noted that the profiles of participants in each group were significantly different, with younger students preferring virtual simulation and students aged 24–33 preferring traditional high fidelity (manikin-based) simulation. The subscales used for the calculations were: Satisfaction with learning, Confidence in learning, Expectations, Perception of high-fidelity simulation, Problem solving, Team collaboration, Active learning, Effectiveness of high-fidelity simulation.

Analyzing the educational level of students subjected to virtual simulation, it was found that those in higher years of study rated their satisfaction with learning, self-confidence, expectations, and overall perception of high-fidelity simulation, as well as problem-solving, active learning, and the overall effectiveness of high-fidelity simulation more positively (Table 8). Expectations, Perception of high-fidelity simulation, Problem solving, Team collaboration, Active learning, Effectiveness of high-fidelity simulation.

Results revealed that women in the traditional high fidelity (manikin-based) simulation group rated their satisfaction with learning and problem-solving significantly higher than men (Table 9). The subscales used for the calculations were: Satisfaction with learning, Confidence in learning, Expectations, Perception of high-fidelity simulation, Problem solving, Team collaboration, Active learning, Effectiveness of high-fidelity simulation.

## Discussion

The aim of this study was to analyze the effectiveness and perception of high-fidelity simulation in a group of medical students by comparing two types of high-fidelity simulation, one using an advanced human simulator and the other a virtual simulation called Body Interact, a software that uses virtual patients in specific scenarios. The study also explored how the two types of training enabled the development of soft skills, such as working in a team or performing precise medical actions under stress.

The study involved 130 medical students completing courses at the Medical Simulation Centre of the University of Rzeszów. The study focused on medical students aged between 19 and 33 years. In the course of the study, respondents were assigned to two groups: group A (*N* = 67) consisted of students performing the selected scenario using the Body Interact virtual patient. Group B (*N* = 63), on the other hand, included students completing the scenario in a traditional high fidelity (manikin-based) simulation. The following research tools were administered following the scenario: The Simulation Design Scale (SDS), the Educational Practices Questionnaire (EPQ), the Student Satisfaction and Self-Confidence in Learning Scale (SSCL) and a proprietary survey questionnaire.

Simulation-based training (SBT) is a teaching method for all medical students and healthcare professionals that uses simulated environments and scenarios to replicate real-world clinical conditions and experiences. The use of high-fidelity simulation enhances the effect of realism by providing excellent conditions for treatment and patient care (29, 30). It facilitates students’ entry into the hospital environment, which represents a seamless transition between the simulator and the real patient, and is a safe and effective tool for developing and improving healthcare skills (31). However, every medical student should systematically acquire knowledge and skills to provide professional patient care (29, 32–34).

The analysis of our results showed that the effectiveness of high-fidelity simulation did not differ for students exposed to both traditional and BI simulation (*p* = 0.355). There were also no significant differences between virtual and traditional high fidelity (manikin-based) simulation when considering the detailed results of the indicated category, i.e., problem solving, team collaboration and active learning (*p* > 0.05). The evaluation of the effectiveness of both traditional and virtual high-fidelity simulation was high, with the mean *M* ranging between *M* = 4.47 and *M* = 4.58. The hypothesis that the use of traditional high-fidelity simulation (manikin-based) would increase effectiveness in terms of teamwork, active learning and problem solving more than virtual simulation was not supported by the outcome analyses conducted. The results obtained are in line with the findings of other authors, which indicate that simulation was an effective teaching method, facilitated the acquisition of knowledge and skills, and influenced the retention of acquired knowledge in long-term memory (30, 32, 33). The above findings are similar to studies that have shown that realistic scenario-based simulation can increase students’ competence and confidence (35). In addition, knowledge gained in the classroom could be applied to basic skills in clinical practice (36).

Simulation-based training is a valuable tool in medical education, benefiting participants at different stages of training, from students to practicing health professionals (37). Findings highlight the importance of integrating simulation into curricula to ensure comprehensive skill development (29). In Poland, medical simulation has for many years been one of the compulsory forms of teaching included in the educational standards for medical faculties (38).

The analysis of our study showed that the perception of high-fidelity simulation did not differ between students exposed to both types of simulation (*p* = 0.289). There were also no significant differences between virtual and traditional high fidelity (manikin-based) simulation when considering the detailed results of the indicated category, i.e., satisfaction with learning, confidence in learning and expectations (*p* > 0.05). The perception score for both types of high-fidelity simulation was high, with a mean *M* ranging from 4.37 to 4.46. The hypothesis that the use of traditional high fidelity (manikin-based) simulation raises the satisfaction, confidence and expectations of the medical students surveyed to a greater extent, in relation to virtual simulation was not confirmed by the study.

Similar results have been reported by other authors, who have shown that it is a meaningful form of clinical teaching and a useful way to train future medical professionals, and that simulation supports the development of clinical skills (39, 40). A multi-center international cohort study of nursing and medical students aimed at analyzing the impact of simulation training using a virtual patient simulator (VPS) and Body Interact software; showed significant improvements in 5/6 elements related to individual learning and 7/7 elements related to curriculum integration according to the students surveyed. The impact of the VPS experience on the perception of simulation in the learning process was similar in both medical faculties (23). In the study by Naggar and Almaeen (41), simulation-based learning was shown to improve clinical skills, ability to remember learning material, clinical decision making and patient communication skills in a group of students. The use of simulation increased satisfaction with learning and confidence in the learning process.

The analysis of the own study shows that the selected variables in terms of gender, age and year of study of the medical students had a moderate or weak effect on effectiveness. Virtual and traditional simulation were rated as equally effective, but younger students preferred virtual simulation, while students age 24–33 preferred traditional high fidelity (manikin-based) simulation. When analyzing the different types of simulation, women rated active learning higher than men in high-fidelity BI simulation. Gender differences between students in their study were described by Nomura et al. In a questionnaire completed by female students before the study, the subscales ‘expectations’ and ‘interests’ scored significantly higher than male students, as did the subscale ‘expectations’ in the final questionnaire (42).

In addition, our study showed that people under 33 years of age rated satisfaction with learning, self-confidence, problem solving, active learning and the overall effectiveness and perception of high-fidelity simulation higher. These observations are consistent with studies by other authors (43, 44).

Our study showed that students in higher years rated satisfaction with learning, confidence, expectations, and overall perception and effectiveness of high-fidelity simulation, as well as problem solving and active learning, higher. This is a natural process related to the knowledge and increasing experience that medical students acquire during their training. The experience and skills that increase with each year of study definitely facilitate working with patients, including simulated patients. In addition, the number of lessons and hours spent on high-fidelity simulation increases with the length of study, which also influences satisfaction and overall perception and efficiency. The findings are consistent with a study in which final-year nursing and midwifery students reported their simulation-based learning experiences as valuable, motivating and confidence-building. All students reported that the simulation-based learning experience enabled them to think more critically about the clinical case scenarios they performed and to critically evaluate their actions and decision-making processes (45).

The last hypothesis assumed that selected variables significantly influence differences in the perception and effectiveness of high-fidelity simulation in a group of medical students. This hypothesis was partially confirmed in the course of the outcome analyses. Results revealed that women in the traditional high-fidelity (manikin-based) simulation group rated their satisfaction with learning and problem-solving significantly higher. Analysis of the virtual simulation BI showed that women rated their satisfaction with learning and problem – solving significantly higher than men; that students aged 24–33 rated their satisfaction with learning, self-confidence, overall perception of high-fidelity simulation, problem-solving, active learning, and the overall effectiveness of high-fidelity simulation more positively; higher years of study rated students’ satisfaction with learning, self-confidence, expectations, and overall perception of high-fidelity simulation, as well as problem-solving, active learning, and the overall effectiveness of high-fidelity simulation more positively.

Medical education using medical simulation has proven to be an approach that significantly improves the educational experience and clinical competence of future healthcare professionals. The various types of simulation currently in use, including high-fidelity simulators, virtual reality and virtual patient environments, standardized patients or hybrid simulations, provide students with a safe and controlled environment in which to practice and refine technical and non-technical skills, ultimately improving patient safety and clinical outcomes. The benefits of simulation include improved skill acquisition, error reduction and the ability to repeat exercises without risk to the real patient (44). Immediate feedback and structured debriefing further enhance the learning process, making simulation an invaluable tool in medical education. However, implementation of the process is challenging as it requires significant financial investment, specialized equipment and trained staff. Despite its potential to provide a highly realistic, safe and reproducible learning environment, simulation cannot fully replace real patients in clinical practice (40). Despite these challenges, numerous case studies and empirical research highlight the effectiveness of simulation compared to traditional methods.

### Limitation

The main limitation of our study was that the majority of the students surveyed who participated in the traditional high fidelity (manikin-based) simulation were fourth-year students and above, while the Body Interact virtual simulation mainly involved second-and third-year students. This is due to the fact that the study was conducted on specific days taking into account the availability of equipment, rooms and based on the schedule of medical students at the Medical Simulation Centre. Another limitation was also the relatively small group of students surveyed. Additionally, the researcher may have inadvertently influenced the study outcomes during the debriefing by unconsciously interacting with the students based on personal expectations or attitudes.

## Conclusion

The results regarding the perception of traditional and virtual simulation among medical students were the same. Medical students’ opinions regarding satisfaction and confidence in the simulations used were high and increased during the educational process. Traditional and virtual simulation showed equal effectiveness in the educational process, with both types of simulation receiving feedback of high effectiveness. Variables such as age, gender or year of study generally did not differentiate medical students’ opinions on the perception and effectiveness of both simulations during the educational process. In contrast, detailed analyses of the high-fidelity BI simulations showed that women rated active learning higher. The students age 24–33 rated higher satisfaction with learning, confidence, overall perception and effectiveness of the high-fidelity simulation, problem solving and active learning. It was found that higher year students rated satisfaction with learning, confidence, expectations and overall perception of high-fidelity simulation higher, as well as problem solving, active learning and overall effectiveness of high-fidelity simulation. In addition, younger students preferred virtual simulation, while students up to the age of 33 preferred traditional high fidelity (manikin-based) simulation. However, it should be borne in mind that in our study the majority of students in the traditional high fidelity (manikin-based) simulation group were fourth-year students and above, which may have influenced the results obtained.

## Data Availability

The original contributions presented in the study are included in the article/supplementary material, further inquiries can be directed to the corresponding authors.
